# Editorial: Plant Foods and Dietary Supplements: Building Solid Foundations for Clinical Trials

**DOI:** 10.3389/fnut.2022.881688

**Published:** 2022-04-11

**Authors:** Barbara C. Sorkin, Susan J. Murch, Connie M. Weaver, Mahtab Jafari

**Affiliations:** ^1^National Institutes of Health (NIH), Bethesda, MD, United States; ^2^Department of Biochemistry and Molecular Biology, University of British Columbia, Vancouver, BC, Canada; ^3^Department of Exercise and Nutritional Sciences, San Diego State University, San Diego, CA, United States; ^4^Department of Pharmaceutical Sciences, University of California, Irvine, Irvine, CA, United States

**Keywords:** natural product, translational research, rigor and replicability, dietary supplement, best practice

Clinical trials are the generally accepted gold standard for querying the safety and efficacy of interventions, but they are time- and cost-intensive. Given their high price, it is critical that each clinical trial advance our understanding to the greatest degree feasible. While it is to be expected that many clinical trials will not reject the null hypothesis given the many differences between preclinical models and humans, as well as between ethnobotanical or even epidemiological and clinical contexts, too often when a clinical trial does not reject the null hypothesis myriad post-trial concerns emerge that it would have been better to resolve pre-trial [e.g., whether a different version of the intervention (dose, formulation, timing, etc.) might have been effective for a slightly different outcome or population] and leave the outcome open to different interpretations. Thus, despite the 2,269 papers on curcumin (or turmeric or curcuminoids) published (in English) in the year ending on November 24, 2021 and the 355 “curcumin” clinical trials listed in clinicaltrials.gov (same search terms, all years), the web site of the US National Institutes of Health's National Center for Complementary and Integrative Health says (https://www.nccih.nih.gov/health/turmeric): *Much research has been done on substances from turmeric, but their health effects remain uncertain*. Similarly large volumes of published research on many other natural products (NP) also shed little light.

With the goals of increasing the yield of clear, evidence-based public health guidance from preclinical, epidemiological and clinical research by increasing the application of good practices to the foundational research as well as to its translation to clinical trials, and of addressing more of those myriad concerns before rather than after the clinical trial, this special edition expands on a 2019 review (1). The papers collected here delve into ways to increase the replicability and clinical relevance and thus also the public health-relevant yields from NP research across the spectrum from chemical characterization to clinical trials.

Increased clarity on which components within a chemically complex NP participate in modulating specific biological outcomes should increase the information gained from clinical trials of these products. A related question is “how much of each key chemical reaches the *in vivo* site of action?” Aspects of this include product stability and replicability, bioavailability and metabolism. Optimization of methods for biochemical characterization and standardization of chemically complex NP are described in this topic by Abraham and Kellogg, Coskun et al., Floyd et al., and Lyu et al..

Interactions among the constituents of chemically complex products may contribute to their biological activities, as highlighted by Seigler et al. Replicability thus requires that we ask “what other, as yet unidentified chemicals may contribute to biological activities of this material?”. Abraham and Kellogg, Coskun et al., and Funk and Schneider describe the utility of untargeted chemical characterization in detecting such constituents. The foregoing highlight the challenges, which Coskun et al. and Wright et al. note are particularly critical for NP used in clinical or translational research; they describe applications of orthogonal methods (leveraging different scientific principles) to increase replicability.

Biologically active product constituents must reach their targets at sufficient concentrations for activity. Lyu et al. describe the importance and development of methods for testing the disintegration and dissolution (D&D) of capsules to be used in clinical trials, while Floyd et al. stress the importance of assessing D&D in biorelevant media representing both fed and fasted conditions; this may avoid the need to develop a novel dosage form for clinical applications.

Floyd et al., Weaver and Hodges, and Wright et al. note the challenge and importance of evaluating absorption and pharmacokinetics for chemically complex products. Heterogeneity—genetic, epigenetic, dietary, etc, among humans, as well as between species—may alter product metabolic rates and the formation of biologically active metabolites (2). Chilton et al. demonstrate the application of several approaches to detect human genetic variants which critically modify metabolic flux and the health effects of food (or other NP), with implications for optimizing clinical trial inclusionary criteria and interpretations.

Floyd et al. describe the challenges of designing pre-clinical studies appropriately for translation to human studies. They highlight the importance of selecting the optimal animal models and dosing regimens for the outcomes of interest and for relevance to the population(s) of interest, and note the importance of considering potential sex differences. Weaver and Hodges note that while stringent inclusionary criteria may decrease the sample size required to provide a reasonable likelihood of avoiding a false positive or false negative result, greater inclusivity may allow greater generalizability. Floyd et al. add that other factors such as circadian rhythm and diet should be documented if not controlled, since they may strongly modulate pharmacokinetics.

Moving further toward translation, issues of safety, optimal dosing regimen, tolerability, ability to mask the intervention in controlled trials, optimal trial population(s) and outcome(s), and regulatory compliance are all critical. Clinical researchers must consider the possibility of “floor effects” for ingredients present in the diet (3), and of “drop in” for supplements available over the counter.

Weaver and Hodges adapted general best practices guidelines for human nutrition randomized controlled trials (4) specifically to plant-based interventions. Both Funk and Schneider and Weaver and Hodges note that while clinical trials are the gold standard for testing efficacy and providing evidence of causality, they should be undertaken only where they address a novel question of substantial public health significance.

Funk and Schneider note that clinical trials based on traditional uses may be less likely to succeed where the effect of interest is more likely to respond to a placebo, and that translation of ethnobotanical research may be complicated by cultural differences in symptomatology as well as in other behaviors or even pharmacogenetics.

Wright et al. provide a description of the development of a clinical trial-ready botanical product which meets requirements for toxic contaminants, is comparable in chemistry and dosing to the preclinical research and traditional products, minimizes participant burden (critical for recruitment, retention and compliance), and provides a good match to the placebo control.

Weaver and Hodges note the importance of compliance with regulatory requirements, including those for data integrity and participant privacy and safety. Transparency must be ensured through pre-study registration of the trial protocol including the statistical analysis plan. A CONSORT [Consolidated Statement on Reporting (Clinical) Trials] checklist provides guidance for reporting herbal interventions (5). Equally essential for transparency, these authors describe the importance of thorough reporting and FAIR (Findable, Accessible, Interoperable, Replicable) data for the advancement of knowledge, as well as for compliance with requirements of funding agencies.

Full adoption of the best practices described by authors in this topic will both increase the value of knowledge gained from translational research using chemically complex NP and increase the utility of NP clinical trial results for improving our understanding of NP effects on human health. Adoption of these practices will provide a more solid foundation for building the evidence base for the use of NP for health.

## Author Contributions

BS and MJ wrote the editorial. SM and CW contributed to the editorial. All authors contributed to planning and editing the topic collection.

## Conflict of Interest

CW is a scientific advisor for Produce for Better Health. The remaining authors declare that the research was conducted in the absence of any commercial or financial relationships that could be construed as a potential conflict of interest.

## Publisher's Note

All claims expressed in this article are solely those of the authors and do not necessarily represent those of their affiliated organizations, or those of the publisher, the editors and the reviewers. Any product that may be evaluated in this article, or claim that may be made by its manufacturer, is not guaranteed or endorsed by the publisher.
